# Dynamic Hyperbolic Tangent PSO-Optimized VMD for Pressure Signal Denoising and Prediction in Water Supply Networks

**DOI:** 10.3390/e27111099

**Published:** 2025-10-24

**Authors:** Yujie Shang, Zheng Zhang

**Affiliations:** College of Engineering Science and Technology, Shanghai Ocean University, Shanghai 201306, China; z-zhang@shou.edu.cn

**Keywords:** water supply network, data denoising, variational mode decomposition (VMD), dynamic particle swarm optimization, pressure prediction

## Abstract

Urban water supply networks are prone to complex noise interference, which significantly degrades the performance of data-driven forecasting models. Conventional denoising techniques, such as standard Variational Mode Decomposition (VMD), often rely on empirical parameter selection or optimize only a subset of parameters, lacking a robust mechanism for identifying noise-dominant components post-decomposition. To address these issues, this paper proposed a novel denoising framework termed Dynamic Hyperbolic Tangent PSO-optimized VMD (DHTPSO-VMD). The DHTPSO algorithm adaptively adjusts inertia weights and cognitive/social learning factors during iteration, mitigating the local optima convergence typical of traditional PSO and enabling automated VMD parameter selection. Furthermore, a dual-criteria screening strategy based on Variance Contribution Rate (VCR) and Correlation Coefficient Metric (CCM) is employed to accurately identify and eliminate noise-related Intrinsic Mode Functions (IMFs). Validation using pressure data from District A in Zhejiang Province, China, demonstrated that the proposed DHTPSO-VMD method significantly outperforms benchmark approaches (PSO-VMD, EMD, SABO-VMD, GWO-VMD) in terms of Signal-to-Noise Ratio (SNR), Mean Absolute Error (MAE), and Mean Square Error (MSE). Subsequent forecasting experiments using an Informer model showed that signals preprocessed with DHTPSO-VMD achieved superior prediction accuracy (R^2^ = 0.948924), underscoring its practical utility for smart water supply management.

## 1. Introduction

Pressure signals in urban water supply networks are susceptible to noise interference from external sources (e.g., sensor electronic noise, electromagnetic interference from pumping equipment) during transmission. Concurrently, the pipeline operation itself generates complex pressure fluctuations originating from physical processes such as random water usage across different time periods, pipeline leakage, valve adjustments, and water hammer effects caused by pump start/stops [1,2]. These valid signal components and noise often overlap in the frequency domain, making it highly challenging to extract useful features from the raw signal. Practically, pipeline pressure signals consist of multiple superimposed frequency bands [3], complicating both signal decomposition and feature extraction. For instance, low-frequency daily periodic signals are often mixed with high-frequency transient signals from water hammer effects. Over-decomposition may fragment component signals and introduce new noise, whereas under-decomposition fails to separate high-frequency noise from valid high-frequency components. The complexity of feature extraction lies in distinguishing meaningful signals from noise [4]. For example, early morning water usage patterns include both actual demand and random user consumption noise, and excessive denoising could inadvertently remove useful information.

Conventional denoising techniques exhibit notable limitations. Wavelet transform methods [5], for instance, require subjective threshold selection and are prone to frequency aliasing [6]. Empirical Mode Decomposition (EMD) [7] is hampered by mode mixing and end effects [8,9]. Although Variational Mode Decomposition (VMD) [10] effectively suppresses mode aliasing, its core parameters [K,α] must be set manually, heavily relying on expert experience [11]. To overcome these issues, research has progressed along several directions. One area involves parameter optimization. Zhang et al. [12] employed Particle Swarm Optimization (PSO) to optimize VMD, enhancing feature extraction and reducing noise interference. Zhao et al. [13] integrated the Heap-Based Optimizer (HBO) with VMD to achieve more precise signal decomposition. Another approach incorporates information entropy as an evaluation criterion. Wang et al. [14] successfully denoised low signal-to-noise ratio signals by combining EMD with Cross Power Spectral Density (CPSD) analysis. Yan et al. [15] proposed a power spectrum entropy optimization mechanism that dynamically adjusts filter parameters by monitoring changes in signal power spectrum entropy, effectively separating noise. Furthermore, hybrid algorithms have been developed to enhance performance. Examples include Mei et al. [16] integrating Genetic Algorithm with Singular Value Decomposition (SVD), Wang et al. [17] establishing a coupled EMD-VMD architecture for data denoising.

Despite these advances, denoising methods tailored for water supply network signals still face considerable challenges, as parameter requirements for VMD vary across different application contexts [18]. For instance, Liu et al. [19] proposed a GWO-optimized VMD joint denoising and localization method, which substantially improves leak detection accuracy under low SNR conditions; however, the computational complexity of the optimization process is high. Zhu et al. [20] introduced a GA-VMD-based denoising method which achieved a positioning error of 2.97% in lab settings, yet did not evaluate whether the denoised signal is suitable for prediction models. Similarly, Zhang et al. [21] proposed SABO-VMD for parameter optimization, but modal screening in high-noise environments still depended on a single metric, risking losing effective components. Jiang et al. [22] developed a hybrid VMD and wavelet threshold method but did not resolve the issue of dynamic parameter adaptation in VMD. Truong et al. [23] applied graph neural networks to predict water network pressure; however, this method requires extensive labeled data and does not consider the potential improvement in prediction accuracy through signal denoising.

Based on the current research, VMD-based denoising for water supply networks remains confronted with three major challenges: (1) poor parameter adaptability, often resulting in either incomplete decomposition of high-frequency features (K too small) or modal redundancy (K too large); (2) a lack of reliable, multi-criteria modal screening; and (3) a lack of integration between denoising and prediction, with insufficient integration between denoising and downstream forecasting tasks.

These challenges directly constrain the performance of data-driven water pressure prediction models, such as the Informer model used in this paper [24]. Such models rely heavily on high-quality, high-fidelity input signals to accurately capture inherent hydraulic dynamic patterns (e.g., daily peak water use and weekly cyclical variations). Specifically, insufficient decomposition can leave high-frequency noise in the effective components, which the prediction model may misinterpret as true water use fluctuations, leading to over fitting and increased prediction error (e.g., overestimating the pressure drop during the morning peak) [11]. Excessive decomposition can introduce spurious, physically meaningless modal components that mask or interfere with key periodic patterns in the pressure signal, weakening the model’s ability to learn long-term, stable temporal dependencies, and thus affecting the accuracy of long-term predictions (e.g., for the next 24 h) [25]. Modal screening based on a single criterion carries a high risk of inadvertently deleting components with low variance contribution but predictive value (e.g., slow-changing components reflecting seasonal variations) [26]. This results in incomplete feature information input to the prediction model and introduces systematic biases.

To address these issues and bridge the gap between “optimal denoising” and “improved forecasting”, this paper proposes a dynamic hyperbolic tangent particle swarm optimization VMD (DHTPSO-VMD) method. This method dynamically adjusts the parameters of the PSO using the hyperbolic tangent function. Using the minimum envelope entropy [27] as the fitness function, the algorithm adaptively searches for the optimal [K, α] combination for VMD. This ensures that the decomposed intrinsic mode functions (IMFs) accurately separate physically meaningful components (such as the 24 h fundamental frequency and the multi-day trend term), providing a pure and well-characterized learning foundation for the forecasting model and fundamentally preventing forecast errors introduced by improper decomposition. A dual-metric screening strategy, using the VCR [26] and CCM [28], accurately identifies and removes noise-dominated IMFs while maximally retaining all predictively useful features. This reduces the input noise interference to the forecasting model and effectively mitigates the risk of overfitting. The dynamic hyperbolic tangent strategy imparts excellent stability and convergence to the optimization process, enabling consistent and reliable denoising results across data from different time periods (e.g., weekdays and holidays). This provides high-quality and stable input signals for the prediction model, avoiding model performance jitter caused by fluctuations in input signal quality.

The experimental validation utilized a pressure dataset spanning two consecutive years, comprising 17,544 hourly samples. DHTPSO-VMD was compared with current mainstream methods (including PSO-VMD, EMD, SABO-VMD, and GWO-VMD) on the same dataset. Experimental results indicate that DHTPSO-VMD outperforms all comparative methods across evaluation metrics during the denoising phase. The mean absolute error (MAE) and mean square error (MSE) are also seriously reduced. Data preprocessed with DHTPSO-VMD achieved optimal performance across all three metrics—R^2^, MAE, and MSE. The R^2^ value increased to 0.948924, significantly surpassing results obtained without denoising and with other comparative denoising methods. These experiments validate the superiority of the proposed method for denoising water supply network pressure data and highlight its significant engineering application value.

## 2. Methods

### 2.1. Variational Mode Decomposition (VMD) Theory

VMD is a variational principle-based signal decomposition method that iteratively searches to decompose an input signal into a series of independent modes $u_{k}$, referred to as intrinsic mode functions (IMFs):

(1) $$u_{k}\left(t\right)=A_{k}(t)\cos\left(\phi_{k}(t)\right)$$

where $\left\{u_{k}\right\}=\left\{u_{1},u_{2},\ldots ,u_{k}\right\}$ represents the K IMF components obtained after decomposition; $A_{k}(t)$ is the instantaneous amplitude; and $\phi_{k}(t)$ is the instantaneous phase.

Each mode $u_{k}\left(t\right)$ undergoes a Hilbert transform to obtain its analytic signal:

(2) $$\left[\left(\delta \left(t\right)+\frac{j}{\pi t}\right)*u_{k}\left(t\right)\right]$$

where $\delta \left(t\right)$ denotes the Dirac delta function.

This analytic signal is then frequency-shifted to baseband via exponential correction:

(3) $$\left[\left(\delta \left(t\right)+\frac{j}{\pi t}\right)*u_{k}\left(t\right)\right]e^{-j\omega_{k}t}$$

where $\left\{\omega_{k}\right\}=\left\{\omega_{1},\omega_{2},\ldots ,\omega_{k}\right\}$ represents the center frequency of the modal components.

The demodulated signal is then Gaussian smoothed to estimate the bandwidth of each mode, as represented in Equation (4).

(4) $${\left\|\partial_{t}\left[\left(\delta \left(t\right)+\frac{j}{\pi t}\right)*u_{k}\left(t\right)\right]e^{-j\omega_{k}t}\right\|}_{2}^{2}$$

Equations (5) and (6) formulate the constrained variational problem for decomposing the pipeline network pressure signal:

(5) $$\underset{\left\{u_{i}\right\},\left\{\omega_{i}\right\}}{\min}\left\{\sum_{k=1}^{K}{\left\|\partial_{t}\left[\left(\delta \left(t\right)+\frac{j}{\pi t}\right)*u_{k}\left(t\right)\right]e^{-j\omega_{k}t}\right\|}_{2}^{2}\right\}$$

Subject to:

(6) $$s.t.\sum_{k=1}^{K}u_{k}\left(t\right)=f\left(t\right)$$

where $f\left(t\right)$ is the original pressure signal. The formulation aims to decompose the signal into K band-limited IMF components whose sum equals the original signal.

To transform this into an unconstrained optimization problem, introduce the Lagrangian multiplier $\lambda \left(t\right)$ and penalty factor  α, constructing the augmented Lagrangian function:

(7) $$L\left(\left\{u_{k}\right\},\left\{\omega_{k}\right\},\lambda \right)=\alpha \sum_{k=1}^{K}{\left\|\partial_{t}\left[\left(\delta \left(t\right)+\frac{j}{\pi t}\right)*u_{k}\left(t\right)\right]e^{-j\omega_{k}t}\right\|}_{2}^{2}+\;{\left\|f\left(t\right)-\sum_{k=1}^{K}u_{k}\left(t\right)\right\|}_{2}^{2}+\langle \lambda \left(t\right),f\left(t\right)-\sum_{k=1}^{K}u_{k}\left(t\right)\rangle$$

where α balances reconstruction accuracy against bandwidth constraints, and $\lambda \left(t\right)$ enforces the reconstruction constraint.

The alternating direction multiplier method (ADMM) is used to solve the augmented Lagrangian problem. The modal components $u_{k}$, center frequency $\omega_{k}$, and Lagrangian multipliers λ are iteratively updated until the convergence conditions are met. The specific steps are as follows:

① Iteratively updating the modal components $u_{k}$:

(8) $${\hat{u}}_{k}^{n+1}\left(\omega \right)=\frac{f\left(\omega \right)-\sum_{i\neq k}u_{i}\left(\omega \right)+\frac{\lambda \left(\omega \right)}{2}}{1+{2\alpha \left(\omega -\omega_{k}\right)}^{2}}$$

where ${\hat{u}}_{k}^{n+1}\left(\omega \right)$ represents the frequency domain representation of the k-th IMF component in the n + 1-th iteration.

② Iteratively updating the modal center frequency $\omega_{k}$:

(9) $$\omega_{k}^{n+1}=\frac{\int_{0}^{\infty }\omega {\left|{\hat{u}}_{k}\left(\omega \right)\right|}^{2}d\omega }{\int_{0}^{\infty }{\left|{\hat{u}}_{k}\left(\omega \right)\right|}^{2}d\omega }$$

③ Update the Lagrange multiplier λ until convergence, and output the IMF component:

(10) $${\hat{\lambda }}^{n+1}\left(\omega \right)={\hat{\lambda }}^{n}\left(\omega \right)+\tau \left(\hat{f}\left(\omega \right)-\sum_{k}{\hat{u}}_{k}^{n+1}\left(\omega \right)\right)$$

where τ is the step size parameter, which controls the convergence rate.

④ Repeat the above steps until the convergence criterion is satisfied:

(11) $$\sum_{k}\frac{{\left\|u_{k}^{n+1}-u_{k}^{n}\right\|}_{2}^{2}}{{\left\|u_{k}^{n}\right\|}_{2}^{2}}<\epsilon$$

where ϵ is the preset convergence threshold. In this paper, the convergence threshold set to $\epsilon =10^{-7}$. The iteration is stopped when the reconstruction error is less than ε after 5 consecutive iterations.

### 2.2. Envelope Entropy Theory

Envelope entropyserves as an indicator for evaluating signal complexity and randomness [29], widely used in signal processing for feature extraction and pattern recognition. The core idea involves performing a Hilbert transform to obtain the envelope signal. The calculation procedure is as follows:

First, the analytic signal is obtained via the Hilbert transform:

(12) $$z_{k}(t)=u_{k}(t)+jH\left\{u_{k}\left(t\right)\right\}$$

where $H\left\{\cdot \right\}$ is the Hilbert transform operator.

The envelope signal is then extracted as:

(13) $$e_{k}(t)=\left|z_{k}\left(t\right)\right|=\sqrt{u_{k}^{2}(t)+{\left(H\left\{u_{k}\left(t\right)\right\}\right)}^{2}}$$

This envelope is normalized to form a probability distribution, with a small perturbation term ϵ added to avoid logarithm of zero:

(14) $$p_{k}(t)=\frac{e_{k}(t)+\epsilon }{\sum_{k=1}^{N}(e_{k}(t)+\epsilon )}$$

The envelope entropy is finally calculated as:

(15) $$E_{p}(u_{k})=-\sum_{k=1}^{N}p_{k}\left(t\right)\log p_{k}\left(t\right)$$

where N is the number of signal sampling points.

Envelope entropy reflects the sparsity of a signal: lower values indicate stronger periodicity and dominant information content, while higher values suggest stronger randomness and noise dominance. This property enables the evaluation of signal decomposition quality and optimal parameter selection. In this paper, we utilize envelope entropy to evaluate the quality of signal decomposition and select the optimal decomposition parameters.

### 2.3. DHTPSO Algorithm

The PSO algorithm, inspired by the foraging behavior of bird flocks [30,31], is a population-based optimization technique. In PSO, particles update their positions and velocities by tracking the individual extreme value ($P_{best}$) and the global extreme value ($G_{best}$). The velocity and position update equations are:

(16) $$v_{i}^{t+1}=v_{i}^{t}+c_{1}r_{1}(P_{i}^{t}-K_{i}^{t})+c_{2}r_{2}(G^{t}-K_{i}^{t})$$

(17) $$K_{i}^{t+1}=K_{i}^{t}+v_{i}^{t+1}$$

where $K_{i}^{t+1}$ is the position vector of particle i during the t+1 iteration; $r_{1}$ and $r_{2}$ are random numbers in the interval [0, 1]; $c_{1}$ is the individual learning factor, and $c_{2}$ is the social learning factor.

To address the tendency of standard PSO towards premature convergence, an inertia weight parameter ω was introduced [32]:

(18) $$v_{i}^{t+1}=\omega \cdot v_{i}^{t}+c_{1}r_{1}(P_{i}^{t}-K_{i}^{t})+c_{2}r_{2}(G^{t}-K_{i}^{t})$$

where ω is the inertia weight.

Although the particle swarm algorithm has been improved, its parameters are often preset and remain fixed during use, making it difficult to adapt to the dynamic parameter requirements for global exploration and local exploitation at different stages.

In this context, scholars have proposed a variety of dynamic parameter adjustment strategies, including linearly decreasing inertia weight strategies [33], random adjustment mechanisms [34], and fuzzy logic-based control methods [35]. In addition, hybrid methods at the structural level are proposed, such as an algorithm that combines the particle swarm optimization (PSO) with the decoupling strategy of the simulated annealing algorithm (SA) [30]. However, these methods have certain limitations [36]: although the linear decreasing strategy is simple, its rate of change is constant. In the early stages of the iteration, the weight may decrease too quickly, weakening the global exploration capability; in the later stages of the iteration, the weight may still be large, affecting the convergence accuracy [33]. It is also unable to simulate the complex nonlinear search behavior during the optimization process. Although the random adjustment strategy can increase the diversity of the population, its randomness may lead to unstable convergence speed and non-reproducible results. Fuzzy logic control performance relies on expert knowledge to design fuzzy rules, which is highly subjective and complex to implement. The hybrid algorithm combining Particle Swarm Optimization (PSO) and Simulated Annealing (SA) has high computational complexity [37].

To address the limitations of the traditional PSO algorithm [34], this paper proposes a Dynamic Hyperbolic Tangent PSO (DHTPSO) algorithm. The hyperbolic tangent function is particularly well-suited for this purpose due to its characteristic properties—namely, smoothness, monotonicity, boundedness, and symmetry about the origin. These properties allow it to naturally model the desired parameter dynamics: exhibiting gradual changes in early iterations to facilitate global exploration, while transitioning to more rapid adjustments in later stages to enable precise local fine-tuning. By leveraging these properties, the proposed method achieves adaptive parameter adjustment within the PSO framework, thereby synergistically enhancing both exploration and exploitation capabilities.

DHTPSO dynamically adjusts the inertia weight ω, individual learning parameter $c_{1}$, and social learning parameter $c_{2}$ through the hyperbolic tangent function. In the early stage of algorithm optimization, the hyperbolic tangent function strategy is adopted to make the inertia weight ω and individual learning parameter $c_{1}$ show a dynamic nonlinear decreasing trend; at the same time, the increasing trend of the hyperbolic tangent curve is used to guide the nonlinear increase in the social learning parameter $c_{2}$, so that the particle swarm algorithm can accurately lock the global optimal solution [38].

This paper uses the characteristics of the hyperbolic tangent curve (domain [−4, 4] [39]) to control the change in the inertia weight ω of the particle swarm algorithm. In the early stage of the search, the function decreases slowly, leaving enough time for the particles to conduct a large-scale global search and avoid falling into the local optimum; in the later stage, the function decreases faster, strengthening the local development ability of the particles, enabling them to conduct a refined search near the potential optimal area discovered in the early stage, accurately locking the global optimal solution of K and α, and avoiding misidentification and convergence uncertainty caused by modal overlap. The inertia weight function is specifically expressed as:

(19) $$\omega =\frac{\omega_{\mathrm{start}}-\omega_{\mathrm{end}}}{2}\tanh(-4+\frac{8\;\times \;(T-t)}{T})+\frac{\omega_{\mathrm{start}}+\omega_{\mathrm{end}}}{2}$$

where $\omega_{\mathrm{start}}$ and $\omega_{\mathrm{end}}$ are the maximum and minimum values of the inertia weight coefficient. In this paper, $\omega_{\mathrm{start}}$ = 0.95 and $\omega_{\mathrm{end}}$ = 0.4 are taken; is the current iteration number, and T is the maximum iteration number.

The individual and social learning factors are given by:

(20) $$c_{1}=c_{1\min}+\frac{\left(c_{1\max}+c_{1\min}\right)}{2}\cdot (1-\tanh(\frac{t}{T}\cdot \ln(\frac{c_{1\max}}{c_{1\min}})))$$

(21) $$c_{2}=c_{2\max}-\frac{\left(c_{2\max}+c_{2\min}\right)}{2}\cdot (1-\tanh(\frac{t}{T}\cdot \ln(\frac{c_{2\max}}{c_{2\min}})))$$

where $c_{1\max}$ and $c_{1\min}$ are the maximum and minimum values of the individual learning factor, and $c_{2\max}$ and $c_{2\min}$ are the maximum and minimum values of the social factor, respectively. The parameters are set as follows: $c_{1\max}$ = 2.0, $c_{1\min}$ = 0.5; and $c_{2\max}$ = 2.0, $c_{2\min}$ = 0.5.

The proposed DHTPSO strategy offers distinct advantages over mainstream dynamic PSO approaches by addressing their core limitations. It overcomes the rigid search dynamics of the Linearly Decreasing Inertia Weight (LDIW) [32] via a nonlinear, adaptive transition that optimally balances exploration and exploitation. Its fully deterministic update mechanism eliminates the performance instability inherent in random adjustment methods [34], ensuring reproducible results. Unlike Fuzzy Logic-Based controllers [35], it requires no complex, subjectively designed fuzzy rules, offering a parameter-driven, rule-free adaptation mechanism. Finally, it enhances performance within the standard PSO framework, circumventing the high computational complexity of hybrid algorithms like PSO-SA [37], making it more suitable for computationally sensitive applications such as ours.

### 2.4. Fitness Function

The core of the DHTPSO-VMD method lies in using the DHTPSO algorithm to automatically identify the optimal VMD parameters—the mode number K and the penalty factor α. The optimization is guided by a fitness function designed to achieve high-quality signal decomposition with minimal noise interference and mode mixing.

The fitness function is constructed based on the principle of minimum envelope entropy, which effectively measures the sparsity and randomness of a signal. A lower envelope entropy value indicates a more periodic and informative component, whereas a higher value suggests dominant noise. To prevent the decomposition from producing modes with overlapping center frequencies (mode aliasing), a penalty term is incorporated.

The unified objective function for the DHTPSO optimization is defined as follows:

(22) J(K,α)K, αmin=∑k=1KEp(uk)+λ·FrequencyOverlap(uk)

where $\mathrm{FrequencyOverlap}(u_{k})$ is the frequency domain overlap penalty term, which is used to prevent modal center frequency aliasing, over-decomposition and fragmentation of effective signal features, and guide the optimization towards mode decomposition that accurately reflects the inherent components of the signal without generating redundancy; and λ is a weighting factor that balance the envelope entropy and the frequency domain constraint.

The frequency domain overlap penalty term is shown in Equation (23):

(23) $$\mathrm{FrequencyOverlap}(u_{k})=\sum_{i<j}I(∆\omega_{\mathrm{ij}}<\epsilon )$$

where $∆\omega_{\mathrm{ij}}=\left|\omega_{i}-\omega_{j}\right|$ represents the modal center frequency difference; $\sum_{i<j}I\left(\cdot \right)$ is the indicator function, and the penalty is triggered when the frequency difference is less than the threshold ϵ.

Apply boundary constraints to the updated $K_{i}$ and $\alpha_{i}$: if they exceed the range of $\left[K_{min},K_{max}\right]$ or $\left[\alpha_{min},\alpha_{max}\right]$, they will be truncated to the boundary value.

The DHTPSO algorithm was configured to search for the optimal parameters within the following predefined ranges:

Mode Number, K:$\left[K_{min},K_{max}\right]$=$\left[2,9\right]$.

Penalty Factor, α:$\left[\alpha_{min},\alpha_{max}\right]$=$\left[200,5000\right]$.

The fitness function for the DHTPSO algorithm is defined by Equation (22). For each particle’s position ($K_{i}$,$\alpha_{i}$), the corresponding integer $K_{int}$=round($K_{i}$) and α are used to perform VMD. The resulting IMFs are then used to calculate the fitness value J(K,α). The algorithm iteratively updates the particle swarm to find the parameter set that minimizes this fitness function.

### 2.5. Modal Screening Strategy

#### 2.5.1. Dual-Criteria Threshold Selection

Following VMD, a dual-criteria screening strategy based on the VCR and the CC is employed to accurately identify and eliminate noise-dominant Intrinsic Mode Functions (IMFs). This approach is grounded in the principle that noise components are characterized by both low energy contribution and a lack of morphological correlation with the underlying physical signal. Relying on a single metric can lead to misjudgment. For instance, physiologically meaningful high-frequency transients (e.g., weak pressure fluctuations induced by water hammer effects) may possess low VCR values yet exhibit a high CC due to their synchronization with system events. Conversely, random noise with moderate energy typically demonstrates a very low CC. By requiring a component to simultaneously satisfy the conditions of “negligible energy contribution” (low VCR) and “insignificant morphological correlation” (low CC), this strategy maximizes noise suppression while effectively preserving critical signal information.

Determination of the VCR threshold (2%): Based on the statistical analysis of 30 sets of pressure signals under the no-leakage condition in Area A of Zhejiang Province, the energy distribution of each IMF was calculated using the Welch power spectrum estimation method [40]. The analysis showed that the energy proportion of the noise component was stably lower than 2.1%, while the energy proportion of all effective signal components was not less than 2%. For further verification, cross-validation was performed on 10 sets of datasets from different water use scenarios such as residential, commercial and industrial. The results showed that when the VCR threshold was 2%, the signal-to-noise ratio (SNR) of the denoised signal reached a peak of 35.7 dB (34.2 dB when VCR = 1% and 33.5 dB when VCR = 3%), confirming that this threshold achieved the optimal balance between noise suppression and signal fidelity.

Determination of the CCM threshold (0.3): First, from a statistical significance perspective, for the large-sample time series data (six months of hourly data, N = 4368) used in the paper, 0.3 is a conservative and stringent criterion that far exceeds the critical value required for significance at α = 0.01, ensuring that any mode below this threshold has a statistically insignificant relationship with the original signal [41]. Second, in the context of signal processing and data analysis, a |r| < 0.3 is widely defined as indicating “low correlation”, signifying that such modes contain minimal effective information and are predominantly noise [42]. This approach, informed by successful applications in related signal processing research [19,29] and validated through pre-experiments, robustly identifies and eliminates noise-dominant modes characterized by broad, disordered spectra.

These thresholds were empirically calibrated for urban water supply pressure signals but can be adjusted for other applications, as discussed in Section 2.5.2.

#### 2.5.2. Application Scenario Adaptation

The proposed dual-criteria framework is inherently flexible, and the thresholds can be adjusted to suit specific application requirements and environmental conditions:

High-Noise Environments (e.g., proximal to pumping stations): In scenarios with elevated background noise, stricter thresholds (e.g., VCR < 1.5%, CCM < 0.25) are recommended to mitigate the risk of noise retention by imposing more stringent requirements on both energy and correlation.

Leak Detection Scenarios: For applications focused on early leak detection, more lenient thresholds (e.g., VCR < 2.5%, CCM < 0.35) are advisable. This adjustment safeguards against the spurious exclusion of incipient leak-induced transient signatures, which often possess low energy (VCR typically between 1.8% and 2.2%) but are critically important, thereby enhancing detection sensitivity.

In summary, the thresholds used in this work were empirically calibrated and validated for the specific context of urban water supply pressure signals.

**Figure 1:**
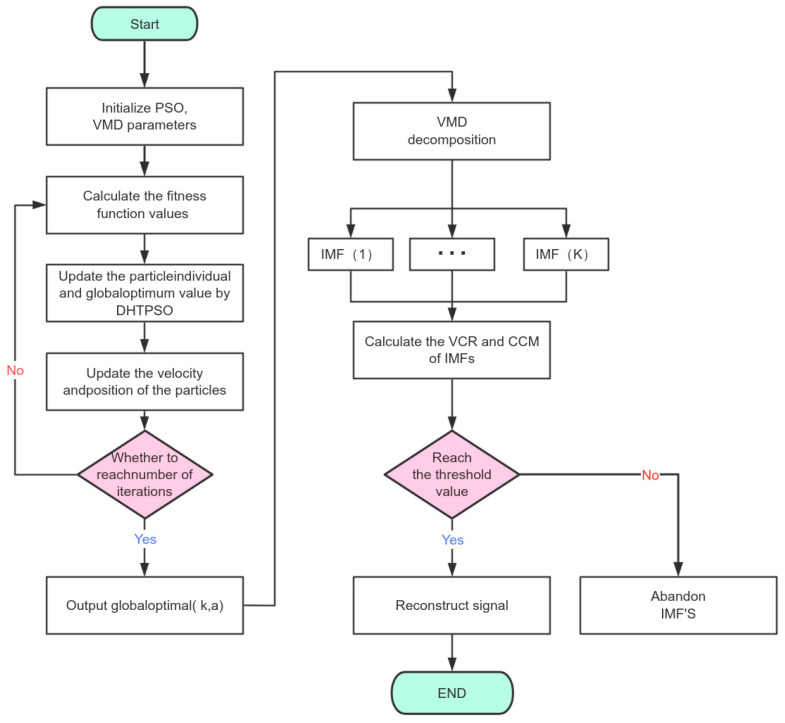
Flowchart of DHTPSO-VMD algorithm for signal denoising.

The flowchart of the DHTPSO-VMD signal denoising algorithm is presented in Figure 1:

## 3. Simulated Experiments

Pressure variations in water supply networks are driven by periodic user consumption behavior, exhibiting pronounced periodic characteristics. The simulation experiment in this paper uses two signals to verify the DHTPSO-VMD denoising method: a noise-free simulation signal (Equation (24)) and a noise-contaminated simulation signal (Equation (25)). The signals contain frequency components at 2 Hz, 60 Hz, and 120 Hz, representing low-, medium-, and high-frequency signals, respectively. The sampling frequency was set to 1000 Hz with a sampling duration of 1 s. Initial parameters for the DHTPSO algorithm were evolutionary generations T = 30, population size N = 15, with inertia weight and learning factors dynamically adjusted using Equations (19)–(21).

**Figure 2:**
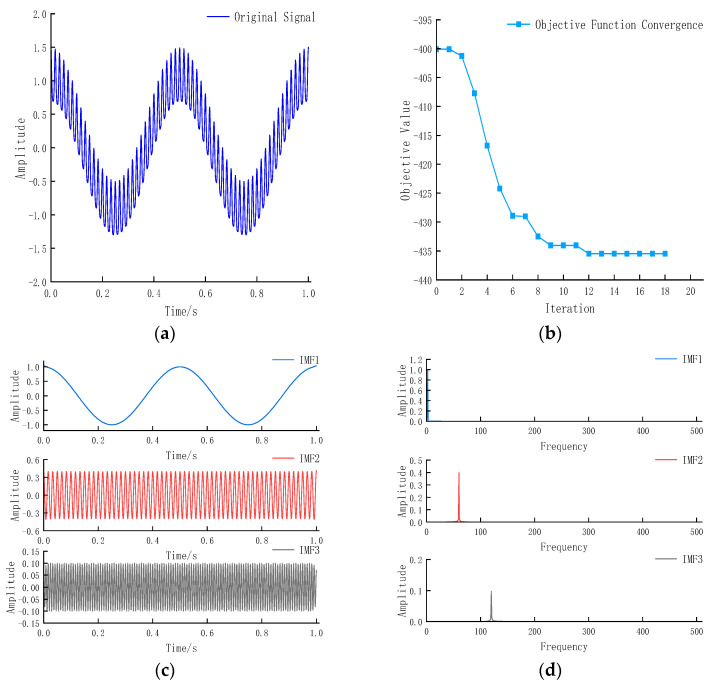
(**a**) shows the original signal; (**b**) convergence curve of objective function; (**c**) IMF components obtained by DHTPSO-VMD decomposition; (**d**) frequency spectrum obtained by DHTPSO-VMD.

The noise-free signal waveform is shown in Figure 2a:

(24) X=cos(2π×2t)+0.4sin(2π×60t)+0.1cos(2π×120t)

VMD was performed on this signal with parameters [K, α] automatically determined by the DHTPSO algorithm. The convergence process of the objective function (Figure 2b) shows the value decreasing from approximately—395 initially, stabilizing at the 12th generation, and finally converging to approximately—435. The most substantial decrease occurred within the first five iterations, indicating rapid approach to the optimal solution region, followed by a local refinement stage with slower decline until convergence at generation 12. The final objective function value stabilized at a low level, confirming successful decomposition into physically meaningful IMF components, thus verifying the effectiveness and robustness of the DHTPSO-VMD method. Figure 2c,d display the decomposed IMF signals and their frequency spectra, respectively, showing consistency with Equation (24) in both amplitude and frequency characteristics, indicating that the signal has been effectively decomposed.

**Figure 3:**
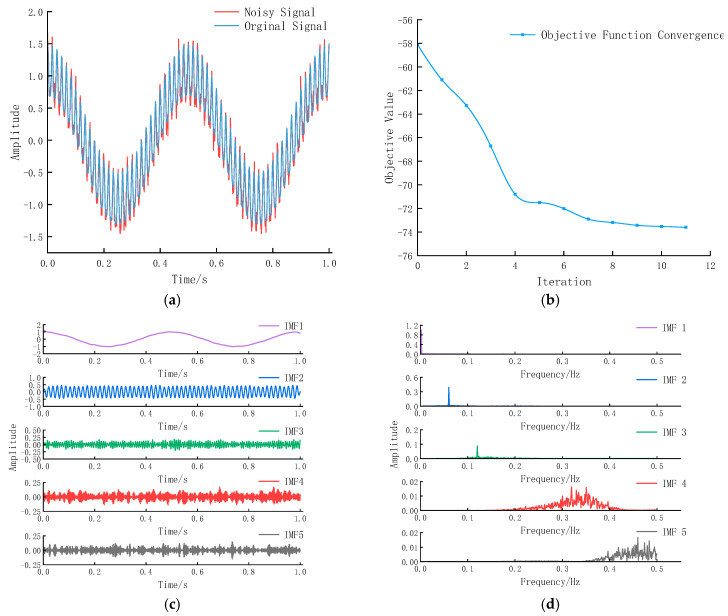
(**a**) Original and noisy signal (σ=0.1); (**b**) convergence curve of objective function; (**c**) IMF components obtained by DHTPSO-VMD; (**d**) frequency spectrum obtained by DHTPSO-VMD.

Random noise η with zero mean and 0.1 standard deviation was added to the simulation signal (24), resulting in the noisy signal (25) shown in Figure 3a:

(25) X=cos(2π×2t)+0.4sin(2π×60t)+0.1cos(2π×120t)+η

The optimization search for the parameters of Equation (25) yielded the optimal VMD parameter combination [K, α] = [5, 438]. The convergence process of the objective function is shown in Figure 3b. VMD using these parameters produced the results shown in Figure 3c,d.

**Figure 4:**
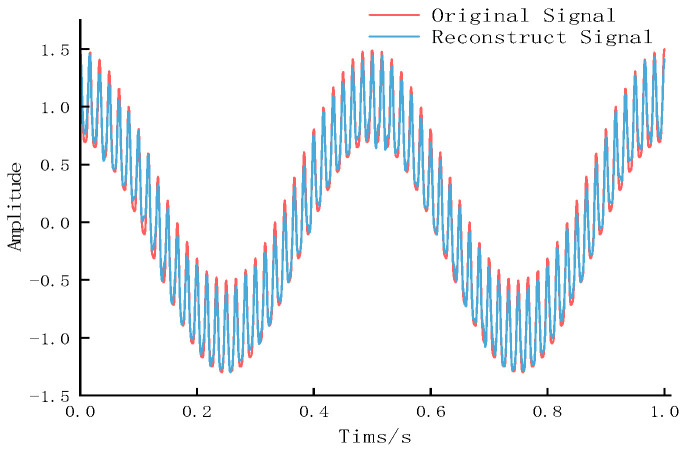
Comparison between the original signal and the reconstructed signal after DHTPSO-VMD denoising.

Table 1 presents the VCR and CCM for each IMF component after DHTPSO-VMD:

As shown in Table 1, IMF4 and IMF5 exhibit low variance contribution rates and correlation coefficients, along with high-frequency random fluctuations, indicating minimal impact on the original signal. These were identified as dominant noise components and removed. The remaining data were reconstructed, with Figure 4 comparing the original signal (Equation (24)) and the denoised reconstructed signal.

To quantitatively evaluate the denoising performance, we calculated the VCR and CCM between the denoised reconstructed signal and the original clean signal (Equation (24)). As shown in Table 2, the signal processed by DHTPSO-VMD exhibits higher VCR and CCM values, closer to 1, than the noisy signal. This indicates that the denoised signal is closer to the original clean signal in both energy and waveform morphology, validating the effectiveness of the method.

The DHTPSO-VMD method demonstrates superior performance in both VCR and CCM compared to the unprocessed signal, confirming its effectiveness in data processing and feature extraction, and validating the optimization of VMD based on the DHTPSO algorithm.

To evaluate the DHTPSO-VMD method’s adaptability to different noise types and intensities, an extended robustness analysis was performed using simulated signals (Equation (24)). The Gaussian noise intensity was increased, and non-Gaussian noise distributions were introduced: (1) Gaussian white noise (standard deviation σ = 0.3); (2) impulse noise with a higher noise ratio and amplitude (15% and 0.8); and (3) Rayleigh distributed noise (scale parameter = 0.3). The VCR and CCM were used as core evaluation metrics to quantitatively analyze the closeness of the noisy signal and the denoised reconstructed signal to the original noise-free signal. The results are shown in Table 3.

Table 3 shows that quantitative evaluations based on VCR and CCM demonstrate that the DHTPSO-VMD method exhibits excellent robustness to various noise types. For high-intensity Gaussian noise (σ = 0.3), the method improves VCR from 0.8523 to 0.9456 and CCM from 0.923547 to 0.972815. Under strong impulse noise (15%, 0.8), VCR and CCM improve to 0.9623 and 0.981036, respectively. Even for non-Gaussian Rayleigh-distributed noise, both metrics significantly improve to 0.9501 and 0.975042. This demonstrates that the method not only effectively handles noise signals of varying intensities and mechanisms, but also effectively preserves the energy and waveform characteristics of the original signal during the denoising process, highlighting its applicability in engineering scenarios.

## 4. Experimental and Results

Pressure data were collected from a water outlet in District A, Zhejiang Province, over two consecutive years from 21 January 2023 to 20 January 2025. This node serves a mixed water use area encompassing residential, hospital, commercial, and industrial users, with annual water consumption of approximately 3.4 million tons. The monitoring point is a municipal water supply main outlet node equipped with Siemens SITRANS P200 pressure sensors (range: 0–1.6 MPa, accuracy: ±0.2%). Data were collected hourly, resulting in 17,544 data points. Missing values were addressed through linear interpolation [43], and outliers were identified and eliminated using the box plot method [44].

The experiment consisted of two parts: first, denoising six months of data (from 21 January to 20 July 2023) to verify denoising effectiveness; second, denoising two years of data (from 21 January 2023 to 20 January 2025) for pressure prediction. DHTPSO algorithm parameters were set as: iterations T = 50, population size N = 30, with inertia weight and learning factors dynamically adjusted using Equations (19)–(21).

### 4.1. Denoising Experiment

**Figure 5:**
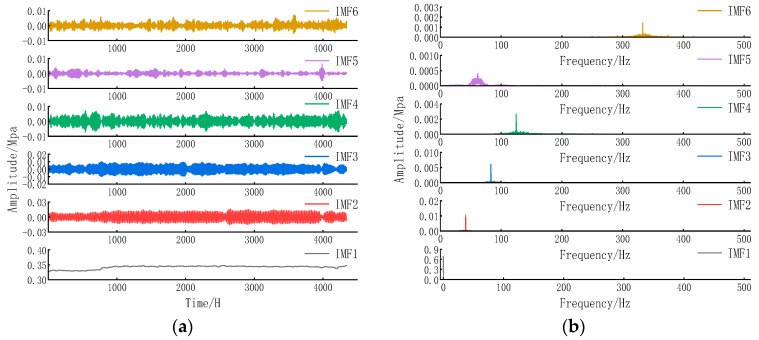
(**a**) VMD of IMF components; (**b**) VMD of and their spectrum.

VMD was performed on data from 21 January to 20 July 2023, with parameters [K, α] automatically determined by the DHTPSO algorithm. The optimal parameters were [6, 3267]. Figure 5a,b show the decomposed IMF signals and their spectra, respectively.

As evidenced by the data in Figure 5, IMF1 and IMF2 exhibit prominent energy signatures and substantial correlation coefficients, identifying them as carriers of dominant periodic hydraulic patterns. Specifically, IMF2 encapsulates the fundamental diurnal (24 h) consumption cycle, while IMF1 reflects modulated oscillations suggestive of multi-day demand trends. IMF3 and IMF4 constitute intermediate components with moderate energy and correlation metrics, potentially corresponding to secondary fluctuations induced by weekly patterns or stochastic consumption events. IMF5 and IMF6 are characterized as noise-dominant modes, evidenced by minimal variance contribution (VCR < 2%), weak correlation (CCM < 0.23), and erratic high-frequency oscillations. Their spectral properties align with stochastic noise originating from measurement artifacts and turbulent flow, justifying their exclusion during signal reconstruction.

Table 4 presents the variance contribution rate and correlation coefficient for each IMF component:

Analysis of Table 4 and Figure 5 indicates that IMF1 possesses strong energy and moderate correlation, capturing main daily pressure fluctuations; IMF2 shows the largest variance contribution and highest correlation, containing the most important pressure change pattern; IMF3 contains relatively weak periodic information, such as monthly or seasonal cycles; IMF4 exhibits low energy, consistent with random water usage information; IMF5 and IMF6 demonstrate variance contribution rates below 2% (0.70% and 1.92%, respectively), indicating negligible energy content consistent with typical low-energy noise characteristics. Physically, these cannot represent effective pressure changes dominated by water usage patterns or pump scheduling. Their correlation coefficients with the original signal are below 0.3 (0.16 and 0.23, respectively), indicating weak correlation and minimal effective information content.

To rigorously evaluate the stability and robustness of the DHTPSO-VMD algorithm, ten independent runs were conducted. The Standard Deviation (SD) is included as a key metric to quantify the dispersion of results across runs, with results shown in Table 5.

The modal number K exhibited strong consistency, converging to the optimal value of 6 in 90% of trials (mean = 6.1, SD = 0.32). This low standard deviation confirms the method’s remarkable reliability in identifying dominant signal components across multiple runs. The single divergent result (K = 7) reflects the algorithm’s effective global exploration capability, successfully escaping local optima. The penalty factor α demonstrated adaptive yet stable behavior, with a mean value of 3244.2 and standard deviation of 268.5. The concentrated distribution of both parameters, especially for the predominant K = 6 cases, underscores the stability and autonomy of the DHTPSO-VMD optimization process.

To determine the optimal decomposition level and validate the parameter selection of DHTPSO-VMD, evaluated the denoising performance under different modal numbers (K = 4, 5, 6, 7). Effective modes were selected using the combined VCR and CCM criteria for signal reconstruction, with SNR, MAE, and MSE calculated as shown in Table 6:

Performance metrics improve consistently as K increases from 4 to 6, achieving optimum at K = 6 with the highest SNR (35.7691 dB) and lowest MAE (4.336 × 10^−3^) and MSE (31 × 10^−6^). This indicates that K = 6 provides sufficient modes to fully separate the signal’s constituent components without information loss. However, increasing to K = 7 leads to degradation across all metrics, indicating over-decomposition beyond the intrinsic number of true components. Thus, K = 6 was identified as the optimal parameter for this dataset.

**Figure 6:**
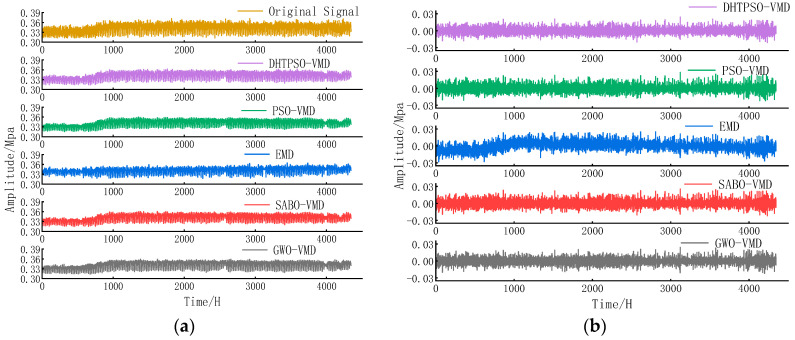
(**a**) Reconstructed signals after denoising by different Denoising methods and (**b**) residual signals after denoising using different Denoising methods.

Using the decomposition results with K = 6 and α = 3267, with IMF5 and IMF6 removed as noise, we then compared the denoising performance of DHTPSO-VMD against PSO-VMD, EMD, SABO-VMD, and GWO-VMD. For PSO-VMD, the inertia parameter was set to 1.5, with both social and individual learning parameters set to 2, using the minimum envelope entropy as the objective function consistent with DHTPSO. Multimodal decomposition was performed on raw pressure data using each method’s optimal parameters. Components with variance contributions below 2% and correlation coefficients below 0.3 were removed before reconstruction. Figure 6 shows the reconstructed and residual signals after denoising using different methods.

Analysis of Figure 6 indicates that DHTPSO-VMD, SABO-VMD, and GWO-VMD perform well in denoising and feature extraction. PSO-VMD shows advantages in preserving detailed information, while the EMD method demonstrates relatively weaker denoising effectiveness.

Signal-to-noise ratio (SNR), mean absolute error (MAE), and mean square error (MSE) were selected as evaluation metrics for denoising effectiveness. To evaluate the stability and robustness of all denoising methods, each algorithm was independently executed 10 times. The resulting performance metrics, presented as mean ± standard deviation (SD), are summarized in Table 7.

The DHTPSO-VMD method achieves an SNR of 35.6172 dB, which is higher than the traditional EMD (32.9830 dB) and superior to GWO-VMD (35.4270 dB), SABO-VMD (35.1101 dB), and PSO-VMD (34.4351 dB). This demonstrates its enhanced ability to preserve effective components of the original signal while suppressing noise. In terms of MAE, DHTPSO-VMD achieves the lowest value (4.287 × 10^−3^), representing reductions of 15.97%, 13.41%, and 10.16% compared to EMD (5.102 × 10^−3^), PSO-VMD (4.951 × 10^−3^), and SABO-VMD (4.772 × 10^−3^), respectively. Furthermore, DHTPSO-VMD also achieves the best MSE performance (30 × 10^−6^), with reductions of 31.82% and 28.57% compared to EMD (44 × 10^−6^) and PSO-VMD (42 × 10^−6^), respectively, demonstrating high reconstruction accuracy and stability.

### 4.2. Prediction Performance Experiment

#### 4.2.1. Data Denoising

**Figure 7:**
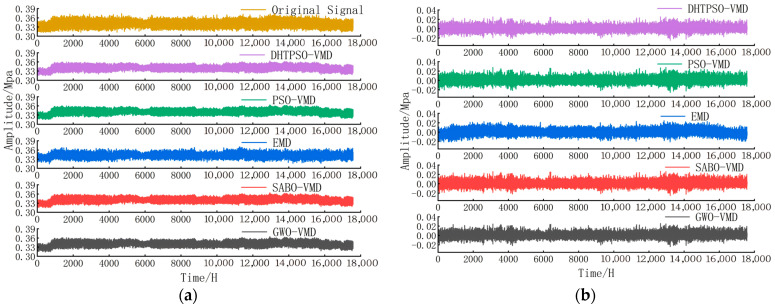
(**a**) Reconstructed signals after denoising by different Denoising methods and (**b**) residual signals after denoising using different Denoising methods.

Validation used using two years of data from 21 January 2023 to 20 January 2025. The reconstructed signals obtained using different denoising methods are shown in Figure 7.

Analysis of Figure 7a reveals that the signal decomposed using DHTPSO-VMD most closely matches the original signal, with highly consistent fluctuation amplitude and morphology. The EMD method performs poorly, showing differences in both amplitude and pattern compared to the original signal, resulting in lower fitting accuracy. Analysis of the residual signals in Figure 7b indicates that DHTPSO-VMD yields the smallest fluctuation amplitude and lowest error; PSO-VMD and GWO-VMD show slightly larger fluctuations, while EMD produces the largest fluctuation amplitude. Overall, DHTPSO-VMD demonstrates superior performance in both signal decomposition accuracy and fidelity.

Table 8 presents the evaluation metrics SNR, MAE, and MSE for the two-year dataset.

The DHTPSO-VMD method achieves an SNR of 35.5698 dB, representing improvements of 4.92%, 8.13%, and 3.20% over PSO-VMD, EMD, and SABO-VMD, respectively, while showing a marginal difference of −0.15% compared to the best-performing GWO-VMD in this metric. In reconstruction accuracy, DHTPSO-VMD yields an MAE of 4.351 × 10^−3^, surpassing PSO-VMD, EMD, SABO-VMD, and GWO-VMD with relative reductions of 20.91%, 19.05%, 15.00%, and 3.07%, respectively. Similarly, it achieves an MSE of 30 × 10^−6^, corresponding to error reductions of 37.50%, 30.23%, 21.05%, and 9.09% compared to the same methods. The consistently superior performance across all three evaluation metrics demonstrates the enhanced denoising capability and robustness of DHTPSO-VMD for high-precision signal processing applications.

#### 4.2.2. Data Prediction

To evaluate the prediction performance and avoid information leakage, a strict temporal split was applied to the two-year dataset (21 January 2023 to 20 January 2025). The dataset was chronologically divided as follows:

Training set: The first 70% of the data.

Validation set: The subsequent 10% of the data. This set was used for hyperparameter tuning of the Informer model and for early stopping to prevent overfitting.

Test set: The final 20% of the data. This set was held out for the final evaluation and comparison of all methods, ensuring that the model was assessed on future data.

Crucially, a strict protocol was followed to prevent data leakage. The complete DHTPSO-VMD optimization process (including parameter optimization and the dual-criteria screening of IMFs) was performed exclusively on the training set to identify the optimal VMD parameters [K, α]. Subsequently, this optimal parameter set was fixed and applied independently to the training, validation, and test sets to perform VMD and dual-criteria modal screening on each dataset. This ensures that the validation and test sets did not, in any form, participate in or influence the parameter optimization, guaranteeing a fair evaluation. This ensures that no future information from the validation or test sets contaminated the denoising process.

Water supply network pressure prediction requires long time-series data input. The probabilistic attention mechanism of the Informer model effectively captures hourly pressure periodicity, making it suitable for this application [24]. The input sequence length (seq_len = 72) corresponds to 72 h (3 days) of historical pressure data. This window is sufficient to capture diurnal patterns (24 h cycles) as well as potential weekly variations (e.g., differences between weekdays and weekends). The prediction length (pred_len = 24) is set to forecast the upcoming 24 h. The label sequence length (label_len = 48) acts as a bridge between the historical context and the prediction horizon, helping the model’s attention mechanism to smoothly transition from known states to future predictions. Other parameters were set as: number of iterations (itr) = 2, and training epochs (train_epochs) = 6.

**Figure 8:**
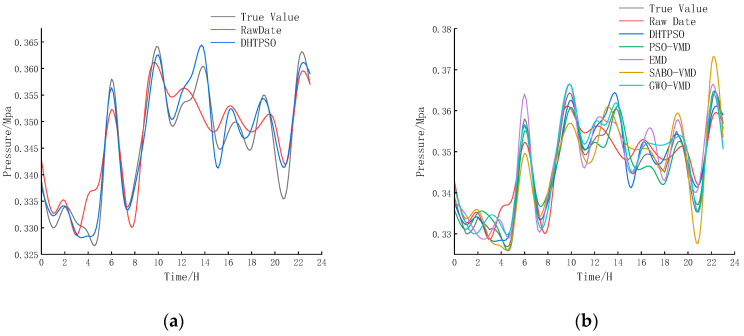
Prediction results after denoising using different methods. (**a**) Prediction results of DHTPSO-VMD denoising method; (**b**) All denoising methods predict results.

Pressure data from Zhejiang District A, denoised and reconstructed by the DHTPSO-VMD algorithm, combined with instantaneous flow data and holiday indicators, formed the input features for the Informer prediction model. To isolate the contribution of the denoising preprocessing, we included a baseline model trained and tested on the raw, un-denoised data. Prediction results are shown in Figure 8.

Analysis of Figure 8 indicates that the prediction model using DHTPSO-VMD denoising achieves high accuracy, while the EMD denoising method shows substantial deviation from actual data, particularly at specific time points. The prediction performance was quantitatively evaluated using the coefficient of determination (R^2^), mean squared error (MSE), and mean absolute error (MAE), as summarized in Table 9.

The prediction performance of all methods is comprehensively compared in Table 9. Compared to the raw data baseline, the proposed DHTPSO-VMD method achieves remarkable improvements: a 49.41% reduction in MAE (from 4.351 × 10^−3^ to 2.201 × 10^−3^), a 75.00% reduction in MSE (from 24 × 10^−6^ to 6 × 10^−6^), and a 17.42% relative improvement in R^2^ (from 0.808152 to 0.948924). These substantial enhancements clearly demonstrate the critical importance of effective denoising preprocessing for pressure prediction tasks.

When compared specifically to other denoising methods, DHTPSO-VMD maintains consistent superiority. It achieves MAE reductions of 35.30% over EMD and 30.98% over PSO-VMD, while the MSE shows even more improvements of 66.67% over EMD and 53.85% over PSO-VMD. The R^2^ value of 0.948924 substantially outperforms both EMD (0.832312) and PSO-VMD (0.882213), indicating stronger explanatory power for data variance.

Furthermore, compared to the more competitive SABO-VMD and GWO-VMD methods, DHTPSO-VMD still demonstrates clear advantages. Relative to SABO-VMD, it achieves improvements of 20.57% in MAE, 45.45% in MSE, and 3.19% in R^2^. Even compared to the second-best performing GWO-VMD, DHTPSO-VMD shows marginal but consistent enhancements across all metrics. These results validate that DHTPSO-VMD preprocessing can effectively identify implicit water usage patterns in supply data while enhancing time series forecasting accuracy, demonstrating strong potential for engineering applications in smart water management systems.

## 5. Conclusions

This paper proposes a novel parameter adaptation strategy utilizing a dynamic hyperbolic tangent function, effectively addressing the tendency of traditional PSO to converge prematurely to local optima while overcoming the empirical parameter selection limitation of VMD. The modal screening strategy based on combined variance contribution rate and correlation coefficient criteria accurately identifies and eliminates noise-dominant IMF components, demonstrating advantages in processing nonlinear and non-stationary signals characteristic of water supply systems. Experimental validation using the Informer prediction model shows that pressure data denoised by this method achieves higher prediction accuracy. Our work provides a valuable methodological framework and technical tool for application scenarios such as water supply network leakage monitoring, energy consumption optimization, and intelligent scheduling.

### 5.1. Limitations

Despite the promising results, the paper has several limitations that should be acknowledged, which also present avenues for future research:

Computational Complexity: The proposed method involves an iterative optimization process, resulting in higher computational complexity compared to some conventional denoising techniques. This may limit its applicability in scenarios requiring real-time processing.

Validation on a Single Node: The proposed method was validated using pressure data from a single outlet node. Its performance and generalizability across water supply networks with different topological structures, pipe materials, and water consumption patterns require further investigation.

Generalization and Robustness: Method performance under different noise distributions (e.g., non-Gaussian or impulsive noise) and with other sensor data types (e.g., flow rate, water quality) requires further assessment. We will validate the framework’s adaptability across diverse operational conditions and data sources.

Comparison with Alternative Models: While the Informer model was demonstrated to be effective, a comprehensive comparison with other state-of-the-art deep learning forecasting models was not conducted to fully situate the performance of the denoising-enhanced pipeline.

### 5.2. Future Work

To address these limitations and advance this research line, our future work will focus on the following directions:

Multivariate extension: In actual water supply networks, there is a close physical coupling relationship between multivariate signals such as pressure, flow, and water level. Future research work will focus on extending this method to collaborative denoising of multivariate signals. A direct and feasible path is to adapt the DHTPSO optimization algorithm proposed in this paper to the dual-criteria screening strategy based on the multivariate variational mode decomposition (MVMD) framework [45]. By mining the synchronized and physically consistent modal components between variables, it is expected to achieve a more robust and hydraulically consistent denoising effect, thereby providing engineering application value for multi-sensor data fusion and state assessment in smart water systems.

Benchmarking against Alternative Forecasting Models: A systematic comparison will be conducted between the Informer model and other advanced time-series forecasting architectures. Specifically, we will evaluate models such as Temporal Convolutional Networks (TCN) [46] for their ability to capture long-range dependencies, and N-HiTS [47] for its hierarchical interpolation-based forecasting strategy, to determine the most synergistic pairing between our denoising method and forecasting models.

Integration with Graph-Based Approaches: We will explore the integration of denoised features with Graph Neural Networks (GNNs) [48]. GNNs can explicitly model the spatial topology and hydraulic connectivity of the water supply network [49], potentially leading to more accurate and physically consistent system-wide pressure predictions.

Algorithm Acceleration for Practical Deployment: To mitigate the computational burden, we will investigate strategies such as surrogate-assisted modeling for the fitness function or parallel computing techniques to accelerate the DHTPSO-VMD algorithm, facilitating its use in real-time monitoring and control applications.

Integration with Advanced Forecasting Models: While Informer was employed here, exploring synergies between denoised features and other state-of-the-art deep learning architectures (e.g., Graph Neural Networks for hydraulic modeling) presents a promising avenue for further improving predictive accuracy.

## 6. Code and Data Availability

To facilitate the reproducibility and extension of this work, we provide the following resources:

Code Repository: The complete implementation of the DHTPSO-VMD algorithm and the experimental framework is publicly available at: https://github.com/Agonist886/DHTPSO-VMD, accessed on 15 October 2025.

Key Implementation Files:

The core denoising algorithm is implemented in MoNi-DP.py.

The pressure prediction is based on the Informer model from the Informer2020-main directory.

Configuration and Random Seeds: The experiments were performed using Python 3.10.9. The repository includes detailed configuration settings specifying all hyperparameters for the DHTPSO optimization (population size, iteration counts, bounds for K and α) and the Informer model (seq_len = 72, label_len = 48, pred_len = 24). To ensure deterministic replication of our results, we have fixed the random seeds for Python’s random, numpy, and torch libraries throughout all experiments. The specific seed values are documented in the source code and configuration files.

Representative Synthetic Dataset: Due to the sensitive nature of operational data from urban water supply networks, the real pressure dataset from District A, Zhejiang Province cannot be shared publicly. However, we have created and provided a representative synthetic dataset that mimics the key characteristics of the real signal. This synthetic dataset is generated using Equation (25) from this paper, with the addition of controlled Gaussian noise (σ = 0.1), replicating the multi-frequency components (2 Hz, 60 Hz, 120 Hz) and SNR conditions analyzed in the paper. Running the provided code on this synthetic dataset is expected to yield denoising and prediction results consistent with those reported in Section 3 (Simulated Experiments).

## Figures and Tables

**Table 1:** Variance contribution rate and correlation coefficient of IMFs.

IMF Component	VCR	CCM
IMF1	0.8482	0.9218
IMF2	0.0096	0.1099
IMF3	0.1296	0.3636
IMF4	0.0047	0.0753
IMF5	0.0042	0.0673

**Table 2:** Evaluation of denoising effect.

Denoising Method	VCR	CCM
Noisy Signal	0.9837	0.991889
DHTPSO-VMD	0.9944	0.997235

**Table 3:** Evaluation table of Denoising Performance under Different Noise Types.

Noise Type & Parameters	Denoising Methods	VCR	CCM
Gaussian (σ = 0.3)	Noisy Signal	0.8523	0.923547
	DHTPSO-VMD	0.9456	0.972815
Impulse (15%, 0.8)	Noisy Signal	0.8945	0.945127
	DHTPSO-VMD	0.9623	0.981036
Rayleigh (Scale = 0.3)	Noisy Signal	0.9123	0.955217
	DHTPSO-VMD	0.9501	0.975042

**Table 4:** VCR and CCM for each IMF component.

IMF Component	VCR	CCM
IMF1	0.188566	0.443531
IMF2	0.389408	0.634659
IMF3	0.131351	0.382228
IMF4	0.037153	0.247471
IMF5	0.007034	0.156927
IMF6	0.019239	0.228781

**Table 5:** Optimization results across ten trials.

Trial	K	α
1	6	3267
2	6	3153
3	6	3277
4	6	3389
5	6	2672
6	6	3136
7	6	3312
8	7	3573
9	6	3276
10	6	3387
Mean ± SD	6.1 ± 0.32	3244.2 ± 268.5

**Table 6:** Performance metrics for different modal numbers.

Modal Num (K)	SNR (dB)	MAE (×10^−3^)	MSE (×10^−6^)
K = 4	31.5343	7.400	82
K = 5	34.6844	4.937	40
K = 6	35.7691	4.336	31
K = 7	35.1894	4.609	35

**Table 7:** Denoising performance evaluation (mean ± SD, n = 10).

Denoising Method	SNR (dB)	MAE (×10^−3^)	MSE (×10^−6^)
DHTPSO-VMD	35.6172 ± 0.1523	4.287 ± 0.045	30 ± 1
PSO-VMD	34.4351 ± 0.2845	4.951 ± 0.088	42 ± 2
EMD	32.9830 ± 0.4236	5.102 ± 0.132	44 ± 3
SABO-VMD [21]	35.1101 ± 0.2218	4.772 ± 0.075	37 ± 2
GWO-VMD [19]	35.4270 ± 0.1832	4.434 ± 0.058	32 ± 1

**Table 8:** Denoising performance evaluation for two-year data (mean ± SD, n = 10).

Denoising Method	SNR (dB)	MAE (×10^−3^)	MSE (×10^−6^)
DHTPSO-VMD	35.5698 ± 0.1634	4.351 ± 0.048	30 ± 1
PSO-VMD	33.9016 ± 0.3125	5.501 ± 0.098	48 ± 3
EMD	32.8962 ± 0.3528	5.375 ± 0.126	43 ± 3
SABO-VMD	34.4671 ± 0.2437	5.119 ± 0.082	38 ± 2
GWO-VMD	35.623 ± 0.1921	4.489 ± 0.061	33 ± 2

**Table 9:** Prediction performance evaluation (mean ± SD, n = 10).

Denoising Method	MAE (×10^−3^)	MSE (×10^−6^)	R^2^
Raw Data	3.674 ± 0.142	22 ± 1	0.808152 ± 0.00745
DHTPSO-VMD	2.201 ± 0.041	6 ± 1	0.948924 ± 0.00192
PSO-VMD	3.189 ± 0.106	13 ± 1	0.882213 ± 0.00468
EMD	3.402 ± 0.138	18 ± 2	0.832312 ± 0.00651
SABO-VMD	2.771 ± 0.082	11 ± 1	0.919541 ± 0.00395
GWO-VMD	2.246 ± 0.057	7 ± 1	0.938318 ± 0.00263

## Data Availability

The data presented in this study are available on request from the corresponding author.
